# Supplementary material for: Content-rich biological network constructed by mining PubMed abstracts
Source: BMC Bioinformatics. 2004 Oct 8;5:147. doi: 10.1186/1471-2105-5-147 (PMC528731; doi:10.1186/1471-2105-5-147)
Supplement: Additional File 2 — The original results of the above study (non-essential files are deleted to keep the file size under the limit set by BMC bioinformatics). [file 1471-2105-5-147-S2.bz2 › chilibotAdditionalFile2/dip10/282ID7744247E1105/html/CDC25A_RAF1.html]

 


 **CDC25A** and **RAF1** 
  
Found 6 abstracts in PubMed, retrieved 6.  
 

 What does Google say? 
 PDF only 
| .edu only 

---

**Interactive relationship** (e.g. stimulation, inhibition, etc)

**Stimulatory relationship**- In NIH 3T3 cells, coexpression of wild type Raf 1  [ **RAF1** ]  and phosphatase inactive  **Cdc25A**  led to a marked increase in Raf 1  [ **RAF1** ]  tyrosine phosphorylation in response to platelet derived growth factor.  Ref: 10373531 Mol Cell Biol, 1999
- Raf 1  [ **RAF1** ] , which is known to bind 14 3 3 proteins, has recently been shown to associate with  **cdc25A**  and to stimulate its phosphatase activity.  Ref: 7644510 Proc Natl Acad Sci U S A, 1995
**Neutral relationship**- Tyrosine phosphorylation of the proto oncoprotein Raf 1  [ **RAF1** ]  is regulated by Raf 1  [ **RAF1** ]  itself and the phosphatase  **Cdc25A** .  Ref: 10373531 Mol Cell Biol, 1999
- Since activation of proteins requires dephosphorylation of particular residues in addition to site specific phosphorylation, the anti apoptotic effect of  **Cdc25A**  might derive from its participation in a multimeric protein complex with phosphoAkt and  **Raf1** , two prominent components of survival pathways.  Ref: 11494150 Oncogene, 2001
- **Cdc25A**  was found to dephosphorylate Raf 1  [ **RAF1** ]  on tyrosines that resulted in a significant decrease in Raf 1  [ **RAF1** ]  kinase activity.  Ref: 10373531 Mol Cell Biol, 1999

**Non-interactive relationship** (e.g. studied together, co-existance, homology, etc.)

- 14 3 3 protein has no effect on the  **cdc25A**  kinase activity of Raf 1  [ **RAF1** ] .  Ref: 7644510 Proc Natl Acad Sci U S A, 1995
- Thus, we investigated whether the  **Raf1** , Mdm2, c Myc,  **Cdc25a**  and Cdc25b proto oncogenes, which are implicated in the ras or p53 or pRb pathways, are alternative oncogenic target genes.  Ref: 10894886 Mutat Res, 2000
- These data suggest that the tyrosine phosphorylation of Raf 1  [ **RAF1** ]  is regulated not only by itself but also by  **Cdc25A** .  Ref: 10373531 Mol Cell Biol, 1999
- Genetic analysis of  **Raf1** , Mdm2, c Myc,  **Cdc25a**  and Cdc25b proto oncogenes in dideoxycytidine and butadiene induced lymphomas in B6C3F1 mice.  Ref: 10894886 Mutat Res, 2000
- In  **cdc25A**  overexpressing cells increased levels of serine 473 phosphorylated Akt were found, which co precipitated with  **Cdc25A**  and  **Raf1** .  Ref: 11494150 Oncogene, 2001
